# A Novel Testis-Enriched Gene *Spata33* Is Expressed during Spermatogenesis

**DOI:** 10.1371/journal.pone.0067882

**Published:** 2013-07-02

**Authors:** Hengling Chen, Minhan Yi, Yue Sheng, Hanhua Cheng, Rongjia Zhou

**Affiliations:** Department of Genetics, College of Life Sciences, Wuhan University, Wuhan, People’s Republic of China; Cardiff University, United Kingdom

## Abstract

With an increasing incidence of male idiopathic infertility, identification of novel genes involved in spermatogenesis is an important aspect for the understanding of human testicular failure. In the present study, we have identified a novel gene *Spata33*, also called as *4732415M23Rik* or *C16orf55*, which is conserved in mammalian species. *Spata33* was predominantly expressed in the postpartum and adult mouse testes at mRNA and protein levels. Its expression was increased during the first wave of the spermatogenesis, indicating that *Spata33* may be associated with the meiotic process. Further immunohistochemistry analysis revealed that *Spata33* was mainly expressed in the spermatocytes, spermatogonia and round spermatids. Its expression was uniformly distributed in the nucleus and cytosol in these germ cells, which was further confirmed by Spata33-tagged with GFP staining in the GC-1 and TM4 cells. These results indicated that *Spata33* was predominantly expressed in the mouse testis and associated with spermatogenesis. Identification and characterization of the novel testis-enriched gene *Spata33* may provide a new route for understanding of spermatogenesis failure.

## Introduction

Approximately 15% of human couples sustain some forms of infertility while nearly half of the causes are male infertility [1], [2]. Genetic aetiologies underlie many cases of testicular failure. As molecular mechanisms controlling male fertility remain poorly understood, the therapeutic approaches to male infertility are not well developed.

Spermatogenesis is a production process of mature sperm from the primordial germ cells (PGCs), which includes spermatogonium proliferation through mitosis, spermatocyte division by meiosis and spermatid differentiation. During embryogenesis, PGCs immigrate to incorporate into the sexually indifferent genital ridge, which then differentiates into gonads. The spermatogonia enter the meiosis cell cycle around postnatal day 10 when preleptotene spermatocytes emerge. The haploid round spermatids first appear between day 18 and 20, and further undergo a series of dramatic morphological transformation during their differentiation into elongated spermatids. Finally, mature sperm cells are produced from the center of the seminiferous tubules in approximately 35 days and a new round is initiated about every 12 days [3], [4], [5].

Spermatogenesis is a complexly regulated process. Approximately one half of total coding genes were expressed in mouse testis [6] and many of the genes are specifically involved or differentially regulated at different stages during spermatogenesis [7]. Mutations of genes on the human Y chromosome are particularly involved in male infertility [8]. The deletions of the two genes *DDX3Y* (DEAD-box RNA helicase Y) and *USP9Y* (the ubiquitin-specific protease 9Y gene), which are located in the azoospermia factor (*AZF*) region on the human chromsome Yq, cause Sertoli cell-only syndrome and spermatogenic arrest [9]. Deleted in azoospermia (*DAZ*) gene, a candidate for azoospermia factor *AZFc*, encodes an RNA-binding protein. It promotes meiosis progression and haploid production and its mutations result in oligozoospermia and azoospermia [10], [11]. The X chromosome is also required for mammalian spermatogenesis [12]. The *DAX1*-deficient mice have X-linked hypogonadotropic hypogonadism and spermatogenic failure [13]. In addition, spermatogenesis is controlled by autosomal genes, including recombination genes (*Msh4* and *Msh5*) [14], [15], apoptosis associated genes (*Bcl-2, Bax*) [16], [17], histones (*Tnp1* and *Tnp2*) [18] and *Dmrt1* [19], [20]. The loss-of-function mutations of these genes might not only cause infertility but also testicular tumors and other related diseases [21].

Some genes have been found to be exclusively expressed in testis and critically related to testis development and spermatogenesis [22], [23], [24], [25]. Protamine 1 and 2 (*Prm1* and *Prm2*) are exclusively expressed in the haploid cells. The protamines substitute for histones in the chromatin of sperm during the last part of the postmeiotic phase and package the sperm DNA into a highly condensed, stable and inactive complex [22]. *Pgk2* gene is specifically expressed in spermatids in most mammals. The restriction of *Pgk2* expression to spermatids can maintain glycolysis in the later stages of spermatogenesis when *Pgk1* expression ceases due to the X chromosome inactivation [22], [26]. *HSPA2* is expressed specifically in spermatogenic cells in mouse. Male mice lacking *HSPA2* are infertile because spermatogenic cell development is arrested at the G2/M phase transition of the meiotic cell cycle and late pachytene spermatocytes are eliminated by apoptosis [22], [27]. Identification of novel testis-specific genes will provide comprehensive understanding of the developmentally regulated mechanisms in spermatogenesis.

In this study, we reported a novel gene *Spata33*, also known as *4732415M23Rik* or *C16orf55*, which is only conserved in mammalian species. This gene is predominantly expressed in testis at both mRNA and protein levels. We have characterized its expression patterns during spermatogenesis. The protein of this novel gene is mainly expressed in spermatocytes, and also in spermatogonia and round spermatids, indicating a potential role of developmental regulation during spermatogenesis.

## Materials and Methods

### Animals

Mice (C57BL) and New Zealand White rabbits were purchased from Animal Biosafety Level III Laboratory of Wuhan University. The animals were treated in accordance with the International Guiding Principles for Biomedical Research Involving Animals. The mouse testes were obtained at different postpartum times (P 2–20 and 35).

### RT-PCR and Quantitative Real-time PCR

Total RNAs were extracted from mouse tissues using the TRIzol (Invitrogen, Carlsbad, CA, USA) according to the manufacturer's instructions. 3 µg RNAs were used as templates for the reverse transcription using the oligomer (dT)_20_ as a primer and 200 U M-MLV reverse transcriptase (Promega, Madison, WI, USA). Primers specific for mouse *Spata33* and *Hprt* were designed. RT-PCR was used to amplify *Spata33* with the primers (P1S, 5′ATGGGCCAGTCGAAAAG3′ and P1A, 5′TTCTGTGTTGTGTACATC3′). *Hprt* was used as an inner control (HP1S, 5′CCTGCTGGATTACATTAAAGCACT3′ and HP1A, 5′GTCAAGGGCATATCCAACAACAAA3′). PCR was performed at 95°C for 5 min, with 22 (*Hprt*) or 28 (*Spata* 33) cycles of 94°C for 30 s, 64°C for 30 s, and 72°C for 1 min, finally 5 min at 72°C in a 20 µL reaction. Amplified products were resolved on a 2% agarose gel. The PCR products were cloned and sequenced. We performed the quantitative real-time PCR experiments using the SuperScript III Platinum One-Step Quantitative RT-PCR system (Invitrogen, Carlsbad, CA, USA) with the primers (P2S, 5′TTTTGTGAGTCGGTCGGGTTA3′; P2A 5′GTGCTCTTCTCCTCCTCTTTCTT3′; HP2S, 5′AAGGACCTCTCGAAGTGTTGGATA3′; HP2A, 5′CATTTAAAAGGAACTGTTGACAACG3′). qPCR was performed at 95°C for 5 min, and 40 cycles of 94°C for 30 s, 64°C for 30 s, and 72°C for 30 s in a 20 µL reaction (StepOne™ Real-time PCR System, Applied Biosystems, Foster City, CA, USA). Values of relative expression normalized to the housekeeping gene *Hprt* are calculated.

### Antiserum Production

A full-length cDNA of the mouse Spata33 was inserted into pET-32a (+) to generate Spata33-his protein (P3S, 5′CGGGATCCATGGGCCAGTCGAAAAG3′; P3A, 5′ CGGAATTCTTATTCTGTGTTGTGTACATC3′). The fusion protein is expressed in bacteria BL21, purified by standard procedures and subsequently used to immunize New Zealand white rabbit intramuscularly. 0.5 ml (about 50 to 100 µg) immunogen is combined with equal volume of Complete Freund's Adjuvant (CFA) initially, then with Incomplete Freund's Adjuvant (IFA) for injections. Blood was drawn from the central ear artery and the titer of antibody was evaluated using Western blot analysis.

### Western Blot Analysis

Proteins were extracted and separated by 15% SDS-PAGE (sodium dodecyl sulfate polyacrylamide gel electrophoresis) and electrotransferred onto PVDF (polyvinylidene fluoride) membrane (Millipore, Bedford, MA, USA). The membranes were blocked with 10% (w/v) low-fat milk in TBST (20 mM Tris-HCl pH 7.5, 150 mM NaCl, 0.1% Tween 20). After incubated in 5% BSA with the polyclonal antibody (1∶500) overnight at 4°C, the membranes were treated with HRP-labeled secondary antibody (Pierce, Rockford, IL, USA) for 1 h at room temperature. Positive bands were detected using the ECL kit (Thermo Scientific, Waltham, MA, USA).

### Immunohistochemistry

Mouse testes were fixed in Tissue Tek® (Sakura, Torrance, CA, USA) and sectioned with a cryostat microtome (CM1850, Leica, Bensheim, Germany). The sections were treated with 0.3% H_2_O_2_ for inhibition of endogenous peroxidase activity for 10 min. Nonspecific sites were blocked with goat serum for 1 h at room temperature. The slides were incubated with the Spata33 polyclonal antibody (1∶100) at 4°C overnight, then by a biotinylated secondary antibody (goat anti-rabbit IgG) for 1 h at room temperature. In a final step, the immune complex was revealed with diaminobenzidine tetrachloride (DAB kit, Boster, Wuhan, Hubei, China) and the sections were counterstained with hematoxylin. The signals were detected using a Leica DMLA light microscope (Leica, Wetzlar, Germany).

### Fluorescent Vector Construction and Cell Transfection

To construct ﬂuorescent vector *Spata33-GFP*, the full length of *Spata33* cDNA was amplified by the specific primers (P4S, 5′CGGAATTCTGATGGGCCAGTCGAAA3′; and P4A, 5′CGGGATCCCGTTCTGTGTTGTGTAC3′) and inserted into pEGFP-N1 via *Eco*RI and *Bam*HI. The GC-1 cells (a mouse spermatogonia cell line) was cultured in DMEM/high glucose medium (Thermo Scientific Hyclone, Logan, UT, USA) and TM4 cells (derived from immature mouse Sertoli cells) were in DMEM/F12 medium (Thermo Scientific Hyclone) supplemented with 10% fetal calf serum (Thermo Scientific Hyclone) at 37°C in 5% CO_2_. The cells were seeded on cover slips and transfected with Lipofectamine transfection reagent (Invitrogen, Carlsbad, CA, USA). 48 hours later, the cells were counterstained with Hoechest33258 (Sigma, St. Louis, MO, USA) and observed by a confocal laser scanning microscope (FV1000, Olympus, Tokyo, Japan).

### Sequence and Phylogenetic Analysis

All Spata 33 protein sequences were aligned using the ClustalW and displayed by the Genedoc software. The phylogenetic relationship was calculated by Neighbor Joining (NJ)methods using PHYLIP (Version 3.68, bootstrap 1000 replications). Protein domains were searched against the Pfam database. The potential post-translational modification sites were predicted by NMT (N-Myristoylation), GPS 2.1 (phosphorylation) (www.expasy.org/tools/), NetNGlyc 1.0 (N-Glycosylation) and NetPhos 2.0 softwares (phosphorylation) (www.cbs.dtu.dk/services/).

## Results

### Identification of the Transcript Variants of the Mouse *Spata33*

**Figure 1:**
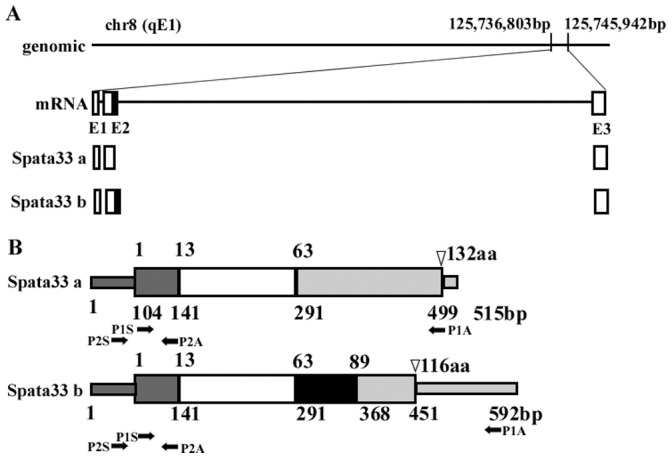
Identification of two transcript variants of the gene *Spata33*. A). Schematic representation of the genomic structure and alternative exons of the gene *Spata33*. Mouse *Spata33* was spliced by alternative 5′ splice site. White blocks represent 3 exons and black block shows alternative exon. B). Schematic view of alternatively spliced transcripts of the *Spata33* gene. Transcript a (GenBank: NM_177279.4) encodes a 132-aa protein. An insertion of expanded exon 2 results in a frameshift, which causes early termination of transcript b (GenBank: CA466259.1). Numbers above the wide rectangles show the amino acid sites, and the numbers below represent nucleotide sites. The locations of the stop codons for each variant are showed in white triangles. Arrows with P1S and P1A indicate RT-PCR primers, and arrows with P2S and P2A denote quantitative RT-PCR primers.

**Figure 2:**
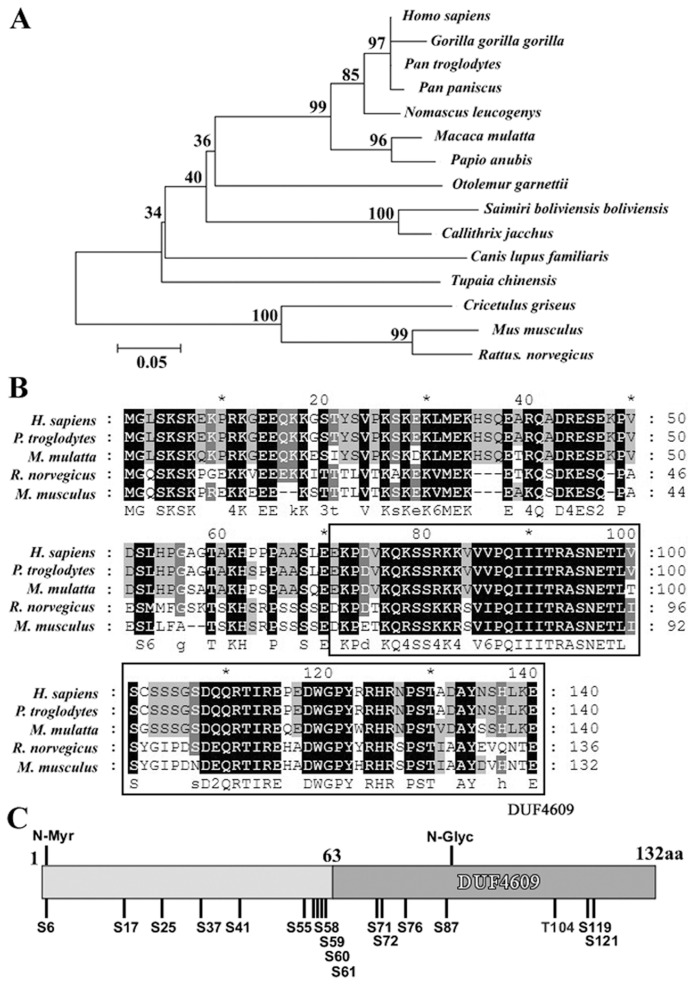
Phylogenetic tree, amino acid alignments, domains and modification sites of Spata33. A). Phylogenetic tree of Spata33 in mammals. Phylogenetic analysis was performed with Phylip. Numbers on the branches represent the bootstrap values from 1000 replicates obtained using the Neighbor-Joining method. The scale bar corresponds to the estimated evolutionary distance units. GenBank accession numbers are as follows: *Callithrix jacchus*, XP_002761326.1; *Canis lupus familiaris*, XP_003434741.1; *Cricetulus griseus*, XP_003495126.1; *Gorilla gorilla gorilla*, XP_004058209.1; *Homo sapiens,* BAG64150.1; *Macaca mulatta,* XP_001104069.2; *Mus musculus*, NP_796253.2; *Nomascus leucogenys*, XP_003280677.1; *Otolemur garnettii,* XP_003800884.1; *Pan paniscus*, XP_003805830.1; *Pan troglodytes*, XP_511172.4; *Papio anubis,* P_003917381.1; *Rattus norvegicus*, NP_001099665.1; *Saimiri boliviensis boliviensis*, XP_003944616.1; *Tupaia_chinensis*, ELW62868.1. B). Alignment of amino acid sequences of the Spata33 proteins. Amino acids that are identical in all these species are shown in white letters on black background. The DUF4609 domain predicted by Pfam is boxed. C). Schematic mapping of potential protein domains and post-translational modification sties. The predicted sites for N-myristoylation, N-glycosylation and phosphorylations in Spata33 were indicated (N-Myr, N-myristoylation site; N-Glyc, N-glycosylation site; S/T, Serine/Threonine phosphorylation sites).

Our previous screening of testis-enriched transcripts through transcriptome-wide analysis of sequence data (the I.M.A.G.E. consortium, http://image.hudsonalpha.org/) has predicted two transcripts of the gene *4732415M23Rik* in mouse testis [28]. This gene is a novel gene, also known as *C16orf55*. According the nomenclature of the HUGO Gene Nomenclature Committee (HGNC) [29], we named it as *Spata33* (*Spermatogenesis associated 33*). To further characterize expression of this gene, we first confirmed the transcript variants of *Spata33* in testis. Sequence analysis showed that the variants were generated by the alternative 5′ splice site (Spata33 a and Spata33 b). The Spata33 b (GenBank: CA466259.1) is 77 bp larger than the Spata33 a (GenBank: NM_177279.4) (Fig. 1A, B). The Spata33 a encodes a predicted protein of 132 amino acids with a molecular weight of about 15 KD, whereas an insertion of an expanded exon in the larger transcript Spata33 b caused a frameshift leading to an early termination codon (Fig. 1B). Further homology search of other vertebrates in GenBank showed several predicted homologues only in other mammals (Fig. 2A). An amino acid alignment revealed that the mouse Spata33 shared a high sequence homology with the mammalian homologues (Fig. 2B). The Spata33 protein showed a novel domain DUF4609 (Domain of Unknown Function 4609) by searching the Pfam database. Sequence analysis indicated that Spata33 was a serine-rich protein. Post-translation modification analysis showed that Spata33 had several potential sites: one N-Myristoylation site, one N-Glycosylation, sixteen Ser phosphorylation sites and one Thr phosphorylation site (Fig. 2C).

### *Spata33* is a Novel Testis-enriched Gene

**Figure 3:**
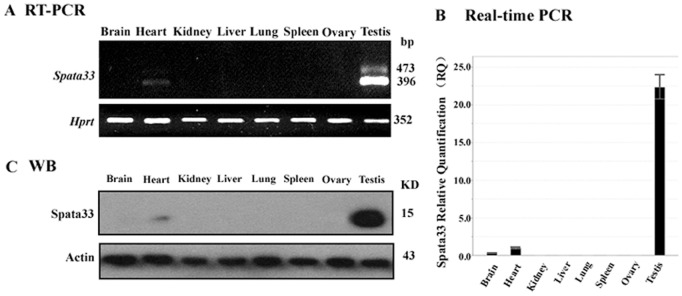
Expression pattern of *Spata33* in various tissues of adult mouse. A). RT-PCR of *Spata33* in adult tissues. *Hprt* was used as a standard. The gene *Spata33* was predominantly expressed in testis. Molecular weight is shown on the right. B). Real-time fluorescent quantitative PCR of the *Spata33* gene in adult tissues. Error bars indicate the standard deviation (SD) of the mean (n = 3). Y-axis represents relative expression levels of *Spata33* and X-axis shows different mouse tissues. C). Expression of Spata33 protein in various tissues. Mouse tissues were subjected to Western blot analysis with antibody against Spata33. Spata33 protein recognized a band at 15 KD. The Spata33 protein was predominantly expressed in testis. Actin was used as an internal control. Molecular weight is shown on the right. We repeated each experiment three times with independent individuals.

To assess expression pattern of *Spata33* in adult mouse tissues, the mRNA level of *Spata33* was examined by semi-quantitative PCR and quantitative real-time PCR in adult tissues. Its expression was markedly high in testis, and very low expression was observed in heart (Fig. 3A,B). To determine its protein level, a polyclonal antibody was raised in rabbit against the coding region of *Spata33*. Western blot analysis showed an unique band at 15 KD in mouse testis, comparable to the predicted molecular weight by Compute pI/Mw [30]. The protein level of Spata33 was consistent with its mRNA (Fig. 3C). These results indicate that the expression of *Spata33* is testis-enriched in mouse.

### *Spata33* is Expressed During Spermatogenesis

**Figure 4:**
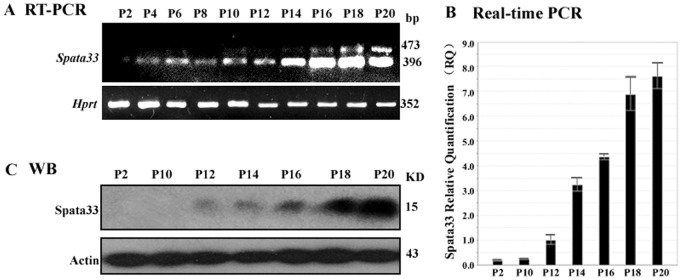
Expression of *Spata33* mRNA and protein in the postnatal testes of mice. A). RT-PCR showed expression pattern of *Spata33* mRNA in testes from P2 to P20 (postnatal days). Total RNAs were isolated from mouse testes and then cDNAs were synthesized. *Hprt* was used as an internal control. Molecular weight is shown on the right. *Spata33* mRNA was increased markedly after P12. B). Real-time fluorescent quantitative PCR of the *Spata33* in testes from P2 to P20. Error bars indicate the standard deviation (SD) of the mean (n = 3). Y-axis represents relative expression levels of *Spata33* and X-axis shows different development stages P2-P20. C). Expression of Spata33 protein in testes from P2 to P20. Mouse testes were individually collected from aged 2 to 20 days and were subjected to Western blot analysis with antibody against Spata33. The protein level was markedly increased from P12. Actin was used as an internal control. Molecular weight is shown on the right. All experiments were performed three times with independent individuals.

We further examined the timing of *Spata33* expression during postnatal testis development in both mRNA and protein levels (Fig. 4A–C). *Spata33* expression was detected in testis soon after birth and reached a high level approximately at 20 days (P20), which is consistent with the first wave of spermatogenesis. Expression of *Spata33* mRNA was weakly detected before P10 and increased steadily from P12 to P20 (Fig. 4A, B). Western blot analysis showed that the Spata33 protein first appeared at P12 and gradually increased from P12 to P20, coincident with the spermatogenia reaching the pachytene diakinesis [4], [5] (Fig. 4C). These results suggest that *Spata33* is developmentally regulated during spermatogenesis.

### Spata33 Protein is Predominantly Expressed in the Spermatocytes

**Figure 5:**
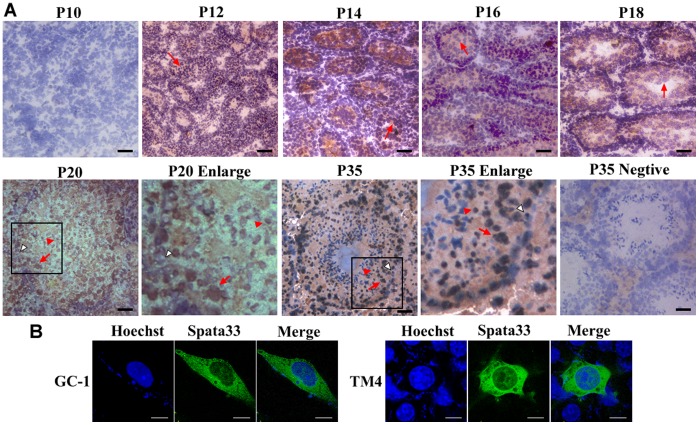
Immunostaining of Spata33 protein in the juvenile (aged 10–20 days) and mature testes (aged 35 days) using anti-Spata33 antibody (A), and subcellular localization of Spata33 protein in the GC-1 cells and TM4 cells (B). A). Spata33 was expressed mainly in the spermatogonia (white arrow heads), spermatocytes (red arrows) and round spermatids (red arrow heads). The signals were observed in both the cytosol and nuclei from P12-P35, whereas no signals were detected in P10. In controls, no positive signals were observed in P35 testis sections when Spata33 antibody was replaced by 1% normal rabbit serum. Nuclei were re-dyed with Hematoxylin (blue). Bars, 20 µm. B). GC-1 and TM4 cells were transfected with Spata33-GFP (green, Excitation 488 nm, Emission 507 nm) and stained with Hoechst (blue, Excitation 352 nm, Emission 461 nm) respectively. Spata33 is localized both in the nuclei and cytoplasm within GC-1 cells or TM4 cells. Bar, 10 µm.

In order to investigate the cell types and cellular localization of the Spata33 protein in testis, we performed immunocytochemical staining on sections of the juvenile and adult mouse testes using the rabbit polyclonal antibody. Spata33 was predominantly expressed in both the nucleus and cytoplasm of the spermatocytes from P12 to adults (Fig. 5A). Positive signals of the Spata33 protein were also detected in the spermatogonia and round spermatids, while Spata33 staining was hardly detectable in elongated spermatids. In addition, the subcellular localization of Spata33 protein in both the cytoplasm and the nucleus was further confirmed in the GC-1 cells and TM4 cells using Spata33 tagged with GFP staining (Fig. 5B). The localization of Spata33 in the germ cell types suggests that Spata33 is in particular associated with spermatogenesis.

## Discussion

Approximately 50% of genes in the mouse genome may be involved in testis development and over 4000 genes may be associated with human male infertility [6], [31]. Molecular mechanisms and detailed functions of most of these genes remain largely unknown. Further studies of male infertility genes will help to development of potential predictive methods and therapeutic strategies.

Addition of the novel gene *Spata33* in the list of spermatogenesis genes is a new progress in male reproduction. The following evidences support association of *Spata33* with spermatogenesis. First, multiple tissue analysis of *Spata33* indicated that it was predominantly expressed in testis, slightly in heart and no detected in other tissues examined. The testis-enriched expression of *Spata33* implied its potential roles in spermatogenesis. Second, there is a close relationship between the expression level of *Spata33* and the first spermatogenic wave in the postnatal testis. RT-PCR and Western blot analysis indicated both the mRNA and protein levels of *Spata33* were increased between P12 and P20 in the juvenile testes. In the previous reports germ cells are known to enter the meiotic prophase around postnatal day 10 and then proceed through a successive processes of the first wave of meiosis during the following 10 days in mouse [4], [5], [32]. So it is noteworthy that the developmentally regulated expression pattern of *Spata33* may be related to spermatogenic events. Last, immunohistochemistry analysis indicated that the expression of Spata33 protein was mainly localized in spermatocytes, spermatogonia, and round spermatids of the seminiferous tubules. These results provided compelling evidences that *Spata33* may play a potential role in spermatogenesis.

Alternative splicing generates protein isoforms, which may exert similar or different functions. It may also result in non-coding mRNAs. Western blot analysis using Spata33 antibody against the full-length protein detected a 15-KD protein encoded by transcript a. However potential 11-KD protein encoded by transcript b was not detected by Western blot analysis. There are several possibilities: Spata33 b does not translate into the short polypeptide; it may be degraded soon after translated because of too short polypeptide, or expressed too low to be detected. Nevertheless, the functions of the transcript b still remain to be studied.

Spata33 is present exclusively in mammals, which suggests that this gene was newly evolved in the lineage of mammals. Spata33 is a Ser-rich protein with many potential phosphorylation sites, which indicates that this protein may exert its roles through kinase signaling during spermatogenesis. Indeed, the phosphorylation in spermatids and dephosphorylation in mature sperms of the sperm-specific histones H1 and H2B are critical for chromatin packing in the sperm nucleus [33], [34]. Testicular protein kinase 1 and 2 (*TESK1* and *2*) phosphorylated specially cofilin at Ser-3, which play vital roles in regulating actin cytoskeletal reorganization [35], [36], [37]. Further studies on Spata33 phosphorylation will help to our understanding of its functions during spermatogenesis. Given an association between Spata33 and spermatogenesis, exact functions of Spata33 and its molecular mechanisms remain to be studied. Further studies on the functions of the novel gene *Spata33* in spermatogenesis will provide insight into male idiopathic infertility.
